# Effectiveness of Induced Hypothermia on the Prognosis of Post-cardiac Arrest Patients: A Scoping Literature Review

**DOI:** 10.7759/cureus.43064

**Published:** 2023-08-07

**Authors:** Ralph Kingsford Rohit, Charu Tibrewal, Naisargi Shrikant Modi, Parth S Bajoria, Prathma Anandbhai Dave, Siddharth Kamal Gandhi, Priyansh Patel

**Affiliations:** 1 Department of Internal Medicine, Dayanand Medical College and Hospital, Ludhiana, IND; 2 Department of Internal Medicine, Civil Hospital Ahmedabad, Ahmedabad, IND; 3 Department of Internal Medicine, Gujarat Medical Education and Research Society, Gandhinagar, IND; 4 Department of Internal Medicine, Medical College Baroda, Vadodara, IND; 5 Department of Internal Medicine, Shri M. P. Shah Government Medical College, Jamnagar, IND

**Keywords:** clinical improvement, preventive medicine, post-cardiac arrest, prognosis, hypothermia

## Abstract

Cardiac arrest (CA) is one of the leading causes of death worldwide. Therapeutic hypothermia (TH) is hypothesized to be a reliable practice for better prognosis in post-cardiac arrest (PCA) patients. Medical subject headings (MeSH) terminology was used to search PubMed Central, Medline, and PubMed databases for articles on the use of hypothermia in PCA patients. We selected various clinical trials, meta-analyses and review articles with complete texts in the English language. PCA syndrome occurs after a CA where the body experiences a state of global ischemia and multi-system dysfunction due to the release of reactive oxygen species (ROS) and inflammatory mediators. Hypothermia slows down enzymatic reactions, reduces free radical production, conserves energy, and prevents the accumulation of metabolic waste products. Delaying the time to initiate targeted temperature management (TTM) increases the mortality of patients, the appropriate temperature for TTM has always been debatable. TTM also has various deleterious effects on various organ systems from shivering, and arrhythmias to life-threatening infections but the risks outweigh the benefits for the patients when hypothermia is introduced in PCA care. Our study compares the different modalities to initiate hypothermia from surface cooling devices to intravascular cooling devices, and the adverse effects of each method compared to another.

## Introduction and background

Cardiac arrest (CA) is caused by the abrupt loss of function of the heart and is one of the leading causes of death worldwide, contributing to more than 350000 deaths each year due to out-of-hospital CA and more than 290000 death every year due to in-hospital CA in the United States (US) [1]. According to the American Heart Association (AHA), more than 500,000 children and adults experience CA in the US and less than 15% survive, this emphasizes the crucial role of cardiopulmonary resuscitation (CPR) and post-cardiac arrest (PCA) care in their survival [1]. Around 80% of CA is estimated to occur in asystole (a non-shockable rhythm), 10% as ventricular fibrillation (a shockable rhythm), and the remaining 10% as electromechanical/valvular defects [2]. Recovery and return of spontaneous circulation (ROSC) are only seen in 30% of the patients who suffered CA and 60% of survivors die due to neurological complications, pulmonary infection, and edema [2,3]. Failure of neurological recovery can be the main cause of death in PCA patients. There has been significant improvement in patients’ cognitive functions after targeted temperature management (TTM) but it also depended on the age of the patient as elderly patients showed significantly less improvement because of reduced neuronal plasticity and impaired neurogenesis [2,4]. PCA syndrome usually occurs after successful CPR and can lead to brain injury, sepsis, and ischemic reperfusion injury due to oxidative stress caused by the generation of reactive oxygen species (ROS), amino acids, and inflammatory products during reperfusion [5].

Hypothermia mainly reduces oxygen consumption of the core organs, reduces free radicals, and protects the cell membrane by preventing intracellular oxidation. It has helped improve the conditions of patients suffering from meningitis, anoxic brain injuries, stroke, and encephalopathy and improved recovery to their healthy state [5,2]. TTM is usually recommended as a resuscitation method in unconscious CA survivors by maintaining the body’s core temperature around 32°C to 36°C for at least 24 hours according to 2020 AHA guidelines or targeted normothermia to control core body temperature of <37.7 °C for at least 72h, with fever prevention in the further course of treatment [6]. Although TTM has shown significant improvement in PCA patients' care, there has been significant debate regarding its use as it carries risks of bleeding, sepsis, shivering, cardiac arrhythmias, and electrolyte and metabolic disturbances [7]. There still remains uncertainty whether the improved outcomes in PCA patients are a result of therapeutic hypothermia (TH) alone or all the other previous steps contributing to the ROSC such as proper mechanical ventilation and oxygenation, treating hypotension, infection control, coronary angiography the reason for better results. In this review, we are going to address the knowledge gap regarding how effective is TH in the prognosis of PCA patients, the different methods that can be used in improving the outcome of the survivor, and how TH affects different systems of our body.

Methodology

We conducted a study under PubMed medical subject headings (MeSH), by including the following keywords "Hypothermia, Induced"[Majr]; and "Heart Arrest"[Mesh] AND "Out-of-Hospital Cardiac Arrest"[Mesh] subsequently Boolean operators “AND” and “OR” were used by merging both the searches narrowed it to 258 results. Further duplicate articles were eliminated by following the inclusion criteria. The search strategies and identified articles according to databases are shown in Table 1.

**Table 1:** Search strategies according to respective databases

Keywords	Database	Results
"Heart Arrest"[majr] AND "Out-of-Hospital Cardiac Arrest"[majr]	PubMed, Medline, PubMed Central	6,922
"Hypothermia, Induced"[majr]	PubMed, Medline, PubMed Central	1,349
"Hypothermia, Induced"[majr] AND ("Heart Arrest"[majr] AND "Out-of-Hospital Cardiac Arrest"[majr]	PubMed, Medline, PubMed Central	258

Inclusion criteria consisted of the free full text of English-language literature published from 2013 to 2023. Further studies were narrowed to only humans and age criteria were set above 18 years, after screening the studies we narrowed it down to 46 results.

## Review

Pathophysiology of PCA syndrome

PCA syndrome is a multisystem organ dysfunction that occurs after a patient is being resuscitated from CA, the body experiences a state of global ischemia. Therapy focused on a specific organ usually compromises other organ systems. PCA syndrome mainly comprises the key components listed below:

PCA brain injury: Cerebral oedema, impaired cerebrovascular autoregulation, post-ischemic neuronal degeneration [8]

PCA myocardial dysfunction: Myocardial stunning, systolic and diastolic dysfunction, myocardial fibrosis [9]

Systemic ischemic-reperfusion injury: Impaired vasoregulation, adrenal suppression, systemic inflammatory response, impaired tissue oxygen delivery, and impaired resistance to infection [8]

During CA the body’s homeostatic mechanism is disturbed causing oxygen to no longer ventilate the lungs and stopping blood circulation throughout the body. During this ischaemic state, metabolic waste products like lactic acid and carbon dioxide start accumulating, this state continues till the ROSC is achieved after CPR. Cerebral ischemia is a consequence of the accumulation of various neurotransmitters leading to neuronal cell death like glutamate and aspartate, the extent of cerebral damage is dependent on the accumulation of these neurotransmitters over time [8,9]. During reperfusion, inflammatory injury starts with mitochondrial damage, and endothelial activation leading to ROS triggering an immune-inflammatory response causing the release of proinflammatory cytokines like tumour necrosis factor-α (TNF-α), interleukins (IL-6, and IL-8), and subsequent complement activation. The high levels of these inflammatory mediators are disrupting the body's protective antioxidant mechanisms and result in the oxidative breakdown of lipids, proteins, and nucleic acids that lead to neuron damage [5,8]. CA often affects the vital body systems leading to hypercapnia/hypocapnia, hypoxemia, hyperglycemia, and hyperthermia leading to a state of shock and further increasing the risk of severe brain injury. TH helps decreases the overall oxygen demand of the body, reduces seizure threshold, reduces cell death, and protects neuronal function [9]. However severe ischemic cerebral injury also affects the body’s thermoregulation mechanism, particularly the hypothalamus which is vulnerable to ischemia and hence the ability to generate heat is limited worsening the prognosis of patients [10].

Role of TTM in PCA care

**Figure 1:**
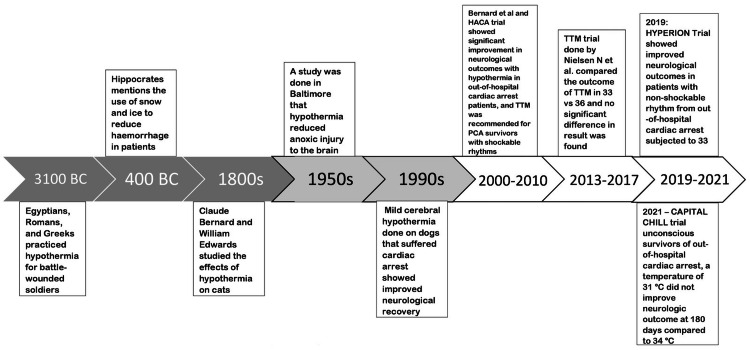
Evolution of therapeutic hypothermia TTM: targeted temperature management, HACA Trial: hypothermia after cardiac arrest trial, PCA: post-cardiac arrest Image credits: Ralph Kingsford Rohit, and Charu Tibrewal

Thermoregulation is defined as the ability of the body to maintain a steady state core temperature between 36.1°C and 37.2°C by balancing heat production and loss [11]. TH can be dated back to ancient Egyptians but its clinical use in hospitals was started around the 1950s when doctors used it in cardiac surgeries to preserve cardiac function and circulation. For every 1°C (1.8°F) decrease in core body temperature, the metabolic rate decreases by about 5% to 7% [12] reducing complications of fever, seizure, and decreasing tissue apoptosis. Cooling the body slows down enzymatic reactions, reduces free radical production, conserves energy, and prevents the accumulation of metabolic waste products [8]. Furthermore, lowering the core body temperature protects against neuronal apoptotic cell death, hypothermia has been proven to relieve cerebral edema by reducing cerebral metabolism and intracranial pressure, improving nerve function, and reducing the degree of injury by improving oxygen supply-demand mismatch [9]. Brain natriuretic peptide (BNP) is a hormone secreted by the ventricles during heart failure. Increased levels of BNP are seen in PCA patients. A study done by Kashiwagi et al. showed that plasma BNP levels significantly increased more than five-fold during TH but heart failure did not worsen; this study can give further insight on improving newer therapeutics under TTM [13]. If a survivor is comatose, TTM should be considered regardless of the initial presenting rhythm; TTM has shown certain cardioprotective effects like preserving left ventricular function, protecting contractility, and preventing microvascular obstruction [14]. Delaying the time to initiate TTM after the ROSC increases the mortality of patients by 20% [15]. A CA survivor bears the risk of neuronal injury via hypoxic-ischemic insult and reperfusion damage, hypothermia mainly preserves high phosphate substrates such as adenosine triphosphate and maintains the pH in the brain [15]. Hypothermia improves neurogenesis, angiogenesis, and gliogenesis after injury; preserves the integrity of the blood-brain barrier and aquaporin channels [14]. Lee et al. in Korea proved that despite the increase in downtime of more than 30 minutes to achieve the ROSC and age less than 70 years, TTM improved neurological outcomes of out-of-hospital CA patients, the effectiveness of hypothermia shouldn’t be ruled out of post-resuscitation care [16]. Even though TH has been a debatable topic; adequate high-quality TTM comprising immediate initiation after anoxic injury, continuous temperature monitoring, use of certain antipyretics drugs to decrease temperature and prevent shivering, selecting appropriate target temperature during the cooling phase, selection of constant temperature during the maintenance phase, a prolonged well-regulated rewarming phase and avoiding pyrexia; following all these steps have significantly improved the outcome of PCA patients [17]. The evolution of TH from ancient times to current times is shown in Figure 1.

Current guidelines for TTM

TTM should be around 33°C-36°C and maintained within a 1°C variation for the duration of therapy. A study conducted in 2013 by Nielsen et al. targeting a core body temperature of 33 °C did not provide any additional benefit compared with a higher core temperature of 36 °C [18]. The TTM-2 study was conducted in 2021 and was the largest study to date, comprising 1800 subjects from around 14 countries and also concluded that TH did not lead to lower mortality than therapeutic normothermia [19]. Due to these studies, many physicians are no longer using TTM to treat patients, as they believe simply keeping them at a normal temperature of 37°C is just as effective without the potential for adverse effects associated with cooling procedures and using sedative drugs.

The introduction of TTM into post-resuscitation care was brought by the hypothermia after CA (HACA) trial around 2002. It showed that post-resuscitation temperature when kept between 32°C and 34°C for 24 hours showed significant neurological improvement compared to normothermia patients in a normal hospital setting. However, the trial also showed a significant mortality risk of pneumonia and sepsis experienced by patients with hypothermia [20]. Additionally, patients subjected to the temperature of 29°C compared to 37°C showed reduced oxidative metabolism, neutrophil chemotaxis, migration, and phagocytosis compared to prior temperatures [21]. Induction of cooling is less efficient in normothermia compared to hyperthermia, at normothermia, the body’s thermoregulatory mechanism maintains a well-maintained target temperature at maximum efficiency however in hyperthermia the central thermostat of the body may be inflamed due to the primary neurological damage [18].

According to AHA 2020, TTM should be kept between 32°C and 36°C for at least 24 hours and also prevent pyrexia in the following 24 to 48 hours after the ROSC [20]. The European Society of Intensive Care Medicine (ESICM) and European Resuscitation Council (ERC) 2022 guidelines recommended continuous monitoring of core body temperature and preventing pyrexia (temperature more than 37.7 °C) in comatose patients/CA patients for 72 hours, also recommended temperature control with antipyretic medicines or cooling devices and prevent the usage of pre-hospital cooling with cold fluid infusion [22]. Australian and New Zealand Committee on Resuscitation (ANZCOR) January 2016 guidelines recommends maintaining a constant temperature of 32-36°C for at least 24 hours and preventing fever in the post-TTM period they did not signify the period of TH in cardiac arrest patients [22]. Beneficiaries from TTM therapy are shown in Table 2.

**Table 2:** Beneficiaries from targeted therapeutic management TTM: targeted therapeutic management, CA: cardiac arrest, CPR: cardiopulmonary resuscitation, ROSC: return of spontaneous circulation

Beneficiaries from TTM Therapy	Non-Beneficiaries from TTM Therapy
Intubated patients and treatment initiated within 6 hours after CA.	Patients who had recent major surgery within 14 days as they carry an increased risk of sepsis and bleeding.
Patients who can maintain a systolic blood pressure above 90 mm Hg, with or without vasopressors (epinephrine), after CPR.	Persistent life-threatening arrhythmias pulseless for more than 60 minutes time since ROSC greater than 12 hours.
Patients (more than 18 yrs) at the time of cooling who remain comatose after CPR and achieving ROSC within 60 minutes of CA.	Coma due to other causes like drug intoxication, pre-existing coma prior to arrest, and status epilepticus.
Unarousable or unconsciousness despite tactile, verbal, and painful stimuli is considered a persistent coma.	Pre-existing blood-related disorders or active bleeding/hemorrhages.

Methods to induce hypothermia

TTM can be divided into three phases: induction, maintenance, and rewarming. The main aim is to target a body temperature between 32°C and 34°C after CA, reduce PCA syndrome complications, further maintain the target temperature for 12-24 hours, and the final phase of controlled rewarming at a rate of 0.2°C to 0.5°C per hour [12].

Induction phase

This is the process of bringing core body temperature to 32°C-34 °C as quickly as possible after CA during ROSC. The induction phase usually starts with the initiation of active cooling till the patient reaches the target temperature [12]. Induction of hypothermia can be initiated in the ambulance for out-of-hospital CA patients by paramedics, this has shown some improvement in prognosis in certain studies. The rapid infusion of cold normal saline (RINSE) trial published in AHA journals found that out-of-hospital CA patients who received cold saline during CPR showed no improvement in prognosis but rather reduced the rate of ROSC with an initial shockable rhythm, forming a question about the time to initiate TTM [23]. Cold intravenous fluid works by decreasing coronary perfusion pressure and increasing central venous pressure which explains the lower rate of ROSC with cold IV fluids and increased adverse effects such as re-arrest and pulmonary edema. However, a trans-nasal evaporative cooling device does not add volume to the cardiac load and reduces brain temperature without reducing the systemic temperature and offering neuroprotection and reducing inflammation [24].

Types of Cooling

Conventional cooling: Followed since ancient times they are mainly cold saline, ice bags, and crushed ice packs were used because they are cost-friendly, have easy availability, easy to use, don’t require expertise, and are a safer method to initiate hypothermia. They can be combined with various other methods of induction to achieve the target cooling temperature efficiently. However, they are not as effective as surface cooling devices (SCD) and intravascular cooling devices (ICD) because they require higher materials, time, and manpower to achieve adequate temperature [10]. Ice packs are placed in the anatomic areas that have large conductivity like the head, neck, axilla, and groin which are replaced when the ice packs are melted making the process messy. The average temperature drop with icepacks is relatively slow about 0.03ºC-0.98°C per hour which is difficult to achieve and maintain target temperature [25]. Thermal heat-exchange cooling pads using water or gel have a better cooling rate (1.33°C per hour for water and 1.04°C per hour for gel) compared with cooling with 30 mL per kg iced saline plus ice packs (0.31°C per hour) [26].

Surface cooling systems: They work by circulating cold fluid or cold air in a blanket or cooling pads applied externally to the patient’s skin. A combination of the water-circulating cooling device and ice packs is effective at maintaining and controlling temperature. They are considered less labour-intensive and easier to use [26]. When equipped with an auto-feedback mechanism that alters the temperature of water or air accordingly to maintain a set target temperature they avoid the risk of overshooting/undershooting the target temperature to extreme hypothermia/hyperthermia. However, they carry a risk of shivering, skin burns, mottling, redness, and dermal irritation [12]. This method also doesn’t require a central venous line as compared to ICD and hence is not time-consuming and does not require constant supervision.

Intravascular cooling techniques: This invasive method includes the placement of a central venous catheter into the internal jugular, subclavian, or femoral vein which maintains temperature by circulating cold saline through a closed loop of the catheter’s balloon. They have a high cooling rate, of 2.0°C-4.5 ºC per hour where large catheters maintain higher temperatures and smaller ones maintain lower temperatures [25,27]. They have been shown to be more effective than surface cooling devices in all three phases of hypothermia; a retrospective study conducted by Glover et al. showed that there was better neurological outcome with ICD compared to SCD which can be due to more precise temperature control with ICD and direct exchange of heat between catheter and blood resulting in rapid transfer of cold blood through the body; compared to SCD where heat conduction occurs slowly through tissue. The clear difference in the mechanism between both methods points out that SCDs have an increased risk of temperature fluctuations; errors in SCDs can lead to intermittent brain hyperthermia [28]. ICDs offer better temperature control during rewarming and less shivering, however, intravascular catheter placement is expensive and carries a risk of thrombosis, insertion-related bleeding, and bloodstream infection which can lead to sepsis and shock [18,14]. A combination of SCD and ICD has shown better results in attaining rapid cooling, maintaining control of target temperature, and minimizing shivering.

Hoedemaekers et al. conducted a study comparing different methods of cooling using water blankets, gel blankets, and intravascular cooling devices in ICU patients and found that all of them are equally effective in inducing hypothermia and normothermia, but ice packs had 60% higher failure to achieve hypothermia or normothermia. They also concluded that ICD is superior at maintaining temperature compared to all other cooling methods [25]. Similarly, the ICEREA trial conducted by AHA showed that both SCDs and ICDs offered clinical value and improvement in prognosis but endovascular cooling compared to basic surface cooling appears to be more efficient in reaching and controlling temperature at 33°C with a decrease in monitoring and workload by the hospital staff and had lesser side-effects [29].

Maintenance phase

The maintenance phase extends from arrival at the goal temperature until rewarming begins, it is equally a pivotal step for better neurological recovery; the cooling phase usually lasts for 24 hours to 48 hours. The maintenance phase requires constant monitoring in order to achieve an accurate target temperature and prevent overshooting or undershooting of temperature [12]. The current method used is the measurement of the temperature of blood using a pulmonary artery catheter considered the gold standard. Other methods used to monitor temperature include bladder, rectum, esophagus, and tympanic membrane sites but they carry a risk of time lag between recorded temperature and actual core temperature due to constant temperature changes in the induction phase [14,12].

Pulmonary artery core temperature although gold standard is an invasive technique and expensive. The closest method which offers accurate results compared to pulmonary artery core temperature is measuring esophageal temperature using a probe of 32-38 cm with an average time lag of three to 10 minutes [26]. However, this method may interfere with certain therapeutic or diagnostic procedures (e.g. intubation, inserting feeding tubes, transoesophageal echocardiography). Another method used commonly in intensive care units (ICU) is the rectal temperature which has a time lag of 15 minutes this procedure is comparatively easier and quick but has a high rate of dislocation [26,27]. Measuring temperature using the bladder has a time lag of 20 minutes, but is efficient as insertion of bladder probe can be combined with catheterization, but this method is less accurate as the rate of urination can fluctuate the temperature hence this is the least commonly used method for temperature measurement [25].

Rewarming phase

**Figure 2:**
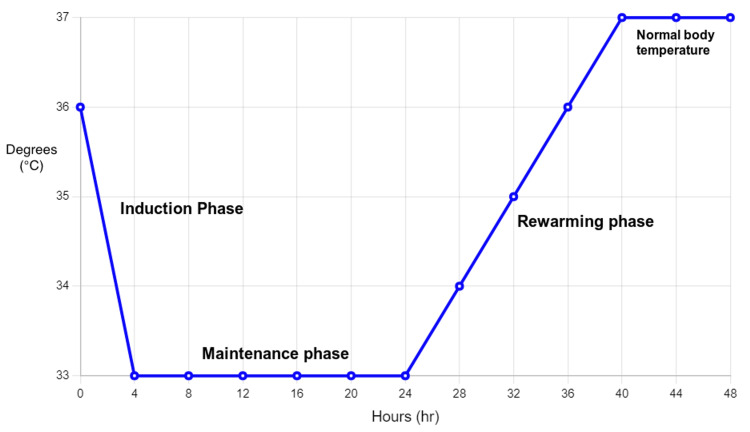
Stages of targeted temperature management Image credits: Ralph Kingsford Rohit, Priyansh Patel, and Charu Tibrewal

This phase starts after cooling where the patient is brought back to normothermia cautiously after hypothermia. During this phase core body temperature is cautiously raised at the rate of 0.15°C-0.25°C per hour until it is higher than 36°C and controlled by rewarming devices as spontaneous rewarming exacerbates neurological injury and can lead to the risk of post-TTM hyperthermia [12]. Duration of rewarming depends on the prognosis of patients and response to TTM during induction, it can start from 12-24 hours after initiating induction and take up to 8 hours [12,14]. The rewarming phase should be done slowly to avoid spikes in intracranial pressure, this will cause hypothermia-induced vasoconstriction to reduce and a change to hypovolemia with hypotension is seen. A prospective study done by Kagawa et al. concluded that prolonged cooling or rewarming for more than or equal to 28 hours did not show a better prognosis but rather led to higher rates of complications like arrhythmia or pneumonia [30]. The relation of temperature change with time from the induction phase to the return of normal body temperature is shown in Figure 2.

Physiological complications of TTM

Shivering

Shivering is the body’s counter-regulatory mechanism in response to hypothermia, shivering increases the body’s metabolic rate and produces heat through rhythmic contraction and relaxation of skeletal muscles, which leads to increased oxygen consumption and energy expenditure [14]. These changes have a negative effect on induced hypothermia as they hinder the beneficial effects and delay the time to achieve the target temperature. Shivering is seen at the time of induction around 35°C to 37°C so patients must be properly sedated with analgesics and antipyretics during this phase and should be stopped only after achieving normothermia [26]. Shivering is usually controlled by both pharmacological and non-pharmacological methods. Nonpharmacological techniques that prevent shivering include wrapping the face, feet, hands, or any exposed area with warm blankets. Pharmacological therapy usually includes short-acting sedatives like propofol, midazolam, or remifentanil to avoid the accumulation of their metabolites which can delay their arousal from coma [31,32]. Magnesium sulfate is also given as they raise the shivering threshold. Antipyretics, α2-agonists (e.g., dexmedetomidine), or neuromuscular blocking agents are also considered to reduce shivering [32].

Cardiovascular and Hemodynamic Complications

The cardiovascular system is commonly affected during TTM from tachycardia and vasoconstriction during the induction phase to bradycardia during rewarming phase. During hypothermia, the body activates its thermoregulatory mechanism and sympathetic system, leading to peripheral vasoconstriction and increased catecholamine production, which can worsen cardiac function by increasing myocardial oxygen demand [12]. Although, therapeutic hypothermia is cardioprotective extreme hypothermia or prolonged hypothermia can lead to coronary vasoconstriction leading to myocardial infarction and life-threatening arrhythmia. The incidence of cardiac arrhythmias increases during hypothermia as renal reabsorption of magnesium is inhibited and potassium shifts intracellularly causing irregular heart rate and rhythm. Such low temperatures are rarely used nowadays, but with methods like SCD without automated control for hypothermia induction when used, the chances of overshooting hypothermia are not uncommon. Sinus bradycardia is the most common conduction abnormality seen during the cooling phase with studies showing prolongation of PR-interval, QTc prolongation, and ventricular escape rhythms [33]. No special attention is usually needed as they resolve during the rewarming phase. Hemodynamic complication like arterial hypotension after ROSC is due to post-resuscitation inflammatory release and global cardiac ischemia. Hypotension should be managed immediately as it can lead to cerebral hypotension and hypoperfusion [14]. Cardiac output decreases during hypothermia and a subsequent increase in systemic vascular resistance is seen; an analytical study done by Bro-Jeppesen et al. on the TTM trial assessing patient parameters at 33°C compared with 36°C for 24 hours observed an increase in systemic and pulmonary vascular resistance index in patients cooled to 33°C versus 36°C, but further cooling below 33°C was associated with a decrease in cardiac index, heart rate, and stroke volume; lactate levels and clearance were also higher in patients cooled to 33°C compared to 36°C [34].

Electrolyte Abnormalities

Many studies observed that hypothermia is associated with diuresis, tubular dysfunction, intracellular ion shift, and a decrease in the concentration of various electrolytes causing hypokalemia, hypomagnesemia, and hypophosphatemia [12]. During rewarming phase, hyperkalemia is often seen due to the release of intracellular potassium and may lead to cardiac arrhythmia. Hypomagnesemia leads to poor neurological outcomes in PCA syndrome patients, hypocalcemia causes reduced contractility, and hypophosphatemia leads to muscle weakness and contractility which can cause ventilator complications [8]. Cold-induced diuresis is a phenomenon caused due to increased atrial natriuretic peptide (ANP) and decreased antidiuretic hormone (ADH) action in the kidney leading to renal tubular dysfunction and increased venous return to the heart leading to vasoconstriction [14]. A randomized control study done on the patients of the TTM trial by Kirkegaard et al. contradicted various trials and showed that hypothermia had less impact on electrolyte levels and urinary excretion and mild change was observed only on electrolytes like potassium, magnesium, and calcium during different phases of TTM. So therefore, regular measurement of electrolytes is necessary to prevent complications in the prognosis and recovery of patients undergoing TTM [35].

Infection

PCA patients are often susceptible to infection due to aspiration, and various invasive methods like (intubation, and catheterization) all can lead to bloodstream infection and compromise the immune system [8]. Hypothermia is known to inhibit inflammatory responses and coagulation factors, reduce pro-inflammatory cytokines, and suppress leukocyte migration and phagocytosis [14]. Patients with prolonged hypothermia are in a hyperglycemic state and are at increased risk of pneumonia sepsis. However adequate study about the effect of hypothermia on microbes and susceptibility to nosocomial infection is yet to be known. Nonetheless, it is necessary to stay vigilant and follow precautions and early identification of infection by routine microbiological profile monitoring, replacing catheters, and inspecting any sites for open wounds due to infections [14,12].

Limitations

The study has limitations regarding knowledge of the appropriate temperature for TTM, and initiation time. Further are needed to evaluate the same. All the studies had very limited clinical trials and had variations in the number of people subjected to TTM, time, age of the patient, and whether the patent had in-hospital cardiac arrest/out-hospital cardiac arrest. The papers reviewed included only papers in the English language and the pediatric population were excluded. Further, the review only includes literature from the past 10 years. Further studies with larger populations and better outcomes are required.

## Conclusions

Although TTM has been implemented in PCA care by many countries, its efficacy is still a question in the medical field. The use of TTM can help in reducing cerebral neuronal injury and inflammation but the question regarding appropriate temperature whether targeted normothermia or hypothermia still requires research and study. Through, this review we can see that TTM therapy affects all the systems including the cardiovascular and central nervous system. Hypothermia reduced cardiac output and work and reduced neuronal injury, cerebral metabolism, and reduced the effects of ROS and inflammatory mediators on various organ systems. Although, hypothermia had positive benefits; over-cooling worsened the mortality of PCA patients leading to arrhythmias, respiratory infection, re-arrest, pulmonary embolism, and death. In some studies, the efficacy of targeted normothermia vs targeted hypothermia saw no benefits with either of the temperature, an optimal temperature for preventing hyperthermia is still required as many prerequisites are needed to achieve a successful TTM therapy. Our review highlighted the importance of TTM in patients and the different methods to induce hypothermia but there are fewer trials regarding the best method to induce hypothermia and reach the appropriate temperature as quickly as possible to reduce the mortality of patients. Finally, in order to achieve a high-quality TTM, a proper method of inducing hypothermia regardless of out-of-hospital CA/in-hospital CA cases; a cross-functional team from paramedics to physicians who need proper understanding regarding their roles is needed to achieve best results in PCA patients.
